# The platelet paradox of injury versus protection in myocardial infarction—has it been overlooked?

**DOI:** 10.1007/s00395-021-00876-6

**Published:** 2021-05-26

**Authors:** Petra Kleinbongard, Ioanna Andreadou, Gemma Vilahur

**Affiliations:** 1 Institute for Pathophysiology, West German Heart and Vascular Center, University of Essen Medical School, University of Duisburg-Essen, Hufelandstr. 55, 45122 Essen, Germany; 2 Laboratory of Pharmacology, Faculty of Pharmacy, National and Kapodistrian University of Athens, Athens, Greece; 3 CIBERCV, Instituto Salud Carlos III, Madrid, Spain; 4 Cardiovascular Research Chair Autonomous University of Barcelona (UAB), Barcelona, Spain

The rupture of an atherosclerotic plaque triggers platelet activation and thrombus formation, often resulting in coronary occlusion and acute myocardial infarction (MI) [3]. Accordingly, the use of antiplatelet therapy has become the standard of care in acute coronary syndromes to attenuate and reduce recurrent thrombotic events. However, the clinical benefit of P2Y_12_ receptor antagonists is not limited to their ability to inhibit platelet function and prevent arterial thrombus formation but they have been shown to also provide direct cardioprotective effects [37, 56].

In the study by *Dr. Hjortbak and collaborators* [23], the authors retrospectively examined data from the CONDI-2/ERIC-PPCI trial. The CONDI-2/ERIC-PPCI trial was an international, multicentre, single-blinded, randomized controlled trial comprising 5401 ST-segment elevation myocardial infarction (STEMI) patients [19]. Patients were randomly assigned to receive either standard treatment or remote ischemic conditioning (RIC), initiated prior to primary percutaneous coronary intervention (PPCI). RIC, brief cycles of ischemia and reperfusion applied to an organ or tissue remote from the heart, has been shown to reduce myocardial damage after myocardial ischemia/reperfusion (IR) in animal models but also in humans [21]. The ability to deliver the cardioprotective RIC stimulus by simply inflating and deflating a pneumatic cuff placed on the upper arm or thigh has facilitated the translation of RIC into the clinical setting [17, 21, 26]. In the CONDI-2/ERIC-PPCI trial, the primary endpoint was a combination of cardiac death or hospitalization for heart failure at 12 months post-randomization. Secondary endpoints included major cardiovascular and cerebral adverse events at 30 days and 12 months and myocardial damage in a subset of 2662 patients estimated from biomarker release over 48 h after PPCI [19]. However, and unfortunately, this trial failed to confirm prior single center studies [11, 20, 22]. There was no difference between the control group and the RIC group in the combined primary endpoint of cardiac death or heart failure or in major cardiovascular and cerebral adverse events or myocardial damage [19].

Now, the group by Dr. Botker has taken advantage of these neutral findings to retrospectively investigate the potential protective effects of the most commonly prescribed P2Y_12_ receptor antagonists—clopidogrel, prasugrel and ticagrelor—on cardiac damage post-MI and clinical outcome [23]. Undoubtedly, this retrospective analysis touches upon a topic of high clinical interest. On one hand, novel and innovative treatment strategies are needed to limit the infarct size, preserve left ventricular function and improve clinical outcomes in STEMI patients undergoing PPCI [2, 10, 18, 33–35]. On the other hand, this multicentre study expands on the reported existence of potential differences in cardioprotection achieved with the most widely prescribed P2Y_12_ receptor antagonists and demonstrates a benefit of ticagrelor over clopidogrel and prasugrel in clinical outcomes. As such, as compared to clopidogrel, the composite endpoint of cardiac death or hospitalization for heart failure was reduced in STEMI patients treated with ticagrelor, but not prasugrel [23]. Platelet P2Y_12_ receptor antagonists are thought to induce cardioprotection through conditioning mimetic effects [45, 56] and thus to potentially limit further protection from additional ischemic conditioning [8, 25]. Yet, an increasing number of experimental and small clinical studies have suggested, that ticagrelor exerts cardioprotection and attenuates adverse cardiac remodelling post-MI to a larger extent than clopidogrel through its platelet-independent effects [1, 38, 51]. In this regard, ticagrelor has been shown to increase the circulating levels of adenosine, an endogenous cardioprotective substance, by inhibiting its uptake through the equilibrative nucleoside transporter 1 receptor [6]. Furthermore, ticagrelor administration has been associated in a pig model of MI with enhanced expression and activation of adenosine monophosphate-activated (AMPK) and reduced aquaporine-4 levels in the ischemic myocardium as compared to clopidogrel-treated animals [52, 53]. In the study by *Dr. Hjortbak* [23], the authors reproduced the clinical data in an in vivo rat model of IR—ticagrelor reduced infarct size, clopidogrel and prasugrel did not. Doses and timing of the P2Y_12_ receptor antagonists were chosen from the literature. However, as discussed by the authors, potential differences in their pharmacokinetic (drug concentrations) or pharmacodynamic (degree of platelet inhibition) profile at reperfusion, that could partly explain ticagrelor’s superiority over prasugrel and clopidogrel, were not excluded. Yet, ticagrelor’s superiority was evidenced in both the preclinical and clinical data. Future studies should aim to determine the cellular targets and signaling pathways by which ticagrelor contributes to such beneficial effects.

**Fig. 1:**
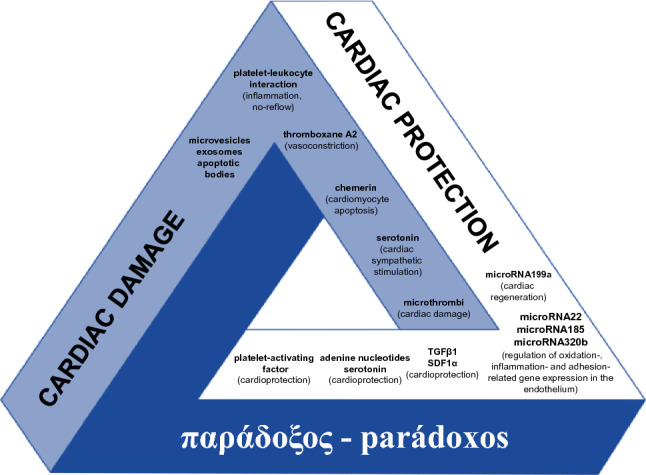
The platelet paradox. Paradox—from the ancient Greek adjective παράδοξος parádoxos [29], “i.e., contrary to expectation, unexpected, incredible”: platelets contribute to cardiac damage during myocardial infarction, but also carry and release multiple cardioprotective factors. Our understanding of platelets in the setting of acute myocardial infarction is not complete—it only appears to be a closed structure—as with the "Penrose triangle". The British Nobel Prize-winning mathematician Sir Roger Penrose popularized this triangle, which gives the appearance of a closed three-dimensional structure of three right angles, but in the Euclidean geometry it cannot exist as a solid object [40]. SDF1α: stromal cell-derived factor-1α, and TGFβ1: transforming growth factor β1

This paper, however, overlooks one crucial aspect: the paradoxical role of platelets in the setting of MI. Activated platelets, beyond promoting arterial thrombus formation, may contribute to cardiac damage via different mechanisms, including the formation of coronary microvascular microthrombi, induction of platelet-leukocyte interactions, release of vasoconstrictor molecules (e.g., thromboxane A2), and (microRNA-containing) microvesicles [4, 9]. However, increasing evidence supports the notion that platelets also carry and release multiple factors with the potential to reduce rather than promote IR injury (Fig. 1) [9, 13]! Whereas the injurious role of platelets is mainly attributed to its (intra-)vascular actions, platelet protective effects are mainly mediated through their secreted factors on cardiomyocytes. Platelets contain sphingosine kinase, which, upon activation, can transform membrane sphingosine into sphingosine-1 phosphate (S1P), a pivotal mediator of cardioprotection [24, 49, 54]. Multiple studies have demonstrated the ability of platelet-derived S1P to protect against IR injury by activating the survivor activating factor enhancement (SAFE), reperfusion injury salvage kinase (RISK), and protein kinase B (Akt)/endothelial nitric oxide synthase cardioprotective signaling pathways [32, 50]. Furthermore, adenosine nucleotides and serotonin released from dense granules have demonstrated tissue-protective effects, and stromal cell-derived factor 1α (SDF1α) and transforming growth factor β1 (TGFβ1) released from alpha granules have been shown to reduce IR injury in rodent and human myocardium [12], and to delay the rate of cardiomyocyte death by activating the RISK and SAFE pathways [46, 55]. Platelets also carry microRNAs, known to exert beneficial effects on cardiac IR, regulate endothelial gene expression and promote cardiac regeneration (Fig. 1) [9]. An in-depth understanding of the role of platelet-derived components on infarct size and cardioprotection in the setting of MI will allow to properly tackle the potential of P2Y_12_ receptor antagonists to limit cardiac damage beyond arterial thrombus formation and growth. It is conceivable that the administration of P2Y_12_ receptor antagonists may interfere with the protective role of platelets during MI [36], making things more complex.

This paper also makes us wonder what the potential impact of RIC on platelet function is. In a recent substudy of the CONDI-2/ERIC-PPCI trial that included 53 patients with RIC versus 47 without, RIC was associated with a reduction in platelet reactivity within the first 48 h post-STEMI [15], confirming a prior single-center trial, where RIC was associated with a reduction in the exercise-related increase of platelet reactivity in patients with obstructive coronary artery disease [5]. RIC has also been shown to prevent systemic platelet activation associated with IR injury in humans [39], to decrease the conformation changes of platelet GPIIb/IIIa (a marker of platelet activation) [28] and platelet–monocyte aggregate formation [27, 28, 48] in patients with suspected stable angina undergoing coronary angiography [27, 28] and in those undergoing ablation for atrial fibrillation [48]. These clinical data support previous experimental findings in animal models of myocardial IR injury in which ischemic conditioning approaches attenuated platelet activation, aggregation and overall platelet-mediated arterial thrombus formation [16, 30]. However, although RIC abrogated the increase in platelet-monocyte aggregation in healthy volunteers, no effect was detected in circulating platelet-neutrophil complexes, and long-term RIC (once/day for 28 ± 4 days) did not alter platelet function in patients with chronic ischemic heart failure [41]. Nevertheless, altogether these data suggest the ability of ischemic conditioning to modulate platelet behaviour in the setting of MI regardless of the antiplatelet background and urges the need to decipher the molecular mechanisms involved, particularly since P2Y_12_ blockade recapitulates protective signal transduction pathways triggered by ischemic conditioning approaches [8, 25]. Future studies should aim to contribute to a better understanding of the basic mechanism(s) of platelet involvement in RIC and investigate in-depth the possible cardioprotective effects of antiplatelet agents that are already used in STEMI patients.

Given the current exceptional situation, a final thought relates to a further platelet paradox in COVID-19 and its current treatment options. SARS-CoV2 is associated with platelet hyperreactivity [7, 14, 43], and some of the vaccination strategies against coronavirus have been suggested to activate platelets [31]. Yet, mounting evidence has indicated that the granular content of platelets plays a critical role in innate immunology in the lung [44], thereby promoting platelet-dependent properties as a therapeutic option for the treatment of COVID-19 [42]. The role of platelets and the impact of antiplatelet approaches [47] in this scenario remain to be explored.

The collaboration of the authors was supported by European COST Action EU-CARDIOPROTECTION COST-ACTION (CA16225).
